# Orodental injury and mouthguard usage in Dutch and international field hockey

**DOI:** 10.4317/jced.61200

**Published:** 2024-02-01

**Authors:** Kirsten E. van Vliet, Jan de Lange, Henk S. Brand, Frank Lobbezoo

**Affiliations:** 1 Dept. of Oral and Maxillofacial Surgery. Amsterdam UMC. University of Amsterdam/Academic Centre for Dentistry (ACTA). Meibergdreef 9, 1105 AZ Amsterdam, the Netherlands; 2 Dept. of Oral Biochemistry. Academic Centre for Dentistry (ACTA). University of Amsterdam and Vrije Universiteit Amsterdam. Gustav Mahlerlaan 3004, 1081 LA, Amsterdam, the Netherlands; 3 Dept. of Orofacial Pain and Dysfunction. Academic Centre for Dentistry Amsterdam (ACTA). University of Amsterdam and Vrije Universiteit Amsterdam. Gustav Mahlerlaan 3004, 1081 LA, Amsterdam, the Netherlands

## Abstract

**Background:**

Mouthguards are used to prevent players from orodental injuries in field hockey. However, such injuries are still a common problem. This study describes the prevalence of orodental injury and the related mouthguard usage in field hockey.

**Material and Methods:**

A 19-item questionnaire was distributed in the Dutch field hockey competition and at the international Master World Cup. In total, 1213 questionnaires were collected. Standard descriptive statistics were used to describe the samples. Associations between data were determined using the Pearson Chi-Square test.

**Results:**

The prevalence of orodental injuries during the career of hockey players was 20% in Dutch players, and 29% in international players. Mouthguard usage among Dutch players was 95%, and among international players 88%. There was no significant association between wearing a mouthguard or not with respect to whether or not treatment was requested as a result of an orodental injury (Dutch *p*=0.43; international *p*=0.22).

**Conclusions:**

This study showed that the prevalence of orodental injuries in field hockey are high, while the majority of the players use a ‘protective’ mouthguard. These results imply that the current mouthguards may not provide enough protection against the forces used in field hockey.

** Key words:**Mouthguard, prevention, sports, dental injury.

## Introduction

Traumatic orodental injuries are not uncommon in ball sports, and do pose serious health risks for many athletes. Among ball sports, field hockey shows a relatively high prevalence of traumatic orodental injuries (1-6). Therefore, hockey players are at risk of irreversible damage of the head and face that can require complete and lengthy restoration with corresponding physical and psychological effects. These injuries may be exacerbated by changes in the aesthetic facial appearance of an individual arising from orodental injuries to areas such as the incisors in the upper jaw. In addition to these direct and indirect consequences of orodental injuries, there is the potential need of further treatment for secondary effects such as root canal problems and their related pain/infection issues and/or the need for dental implants (7). These comprehensive dental treatments also involve high costs.

To reduce orodental injuries in field hockey, organizations around the world are focusing and developing strategies for injury prevention, such as with the recommendation of an important piece of equipment: mouthguards. In general, there are three types of mouthguards: prefabricated mouthguards, which has a standard U-shape form, boil-and-bite mouthguards, which are made from thermoplastic material so a player can mold and form the mouthguards to his/her teeth when heated in water, and custom-made mouthguards, which are fabricated by a professional from a dental impression. Mouthguards claim to distribute the force of impact towards the surrounding structures and can reduce the risk of fractures or avulsion of the teeth (8). Therefore, many studies examined the effectiveness of different mouthguards to prevent or minimize orodental injuries in field hockey (9-15). Consequently, in 2015, the Netherlands implemented that players are mandatory to wear a mouthguards in the field hockey competition. Six years later, the effects of this strict regulation have been evaluated by Cicek *et al*. commissioned by the Dutch Royal Hockey Association (KNHB) (16), who compared the data intern to the previously performed study from Vucic *et al*. (17). That study founded a prevalence of 16% orodental injuries among 1299 Dutch players before this implementation (17). The study from Cicek *et al*. showed that the percentage of respondents wearing a mouthguard during a match increased from 81% before implementation to 91% after implementation of the regulation. However, this study still showed a 6% prevalence of orodental injuries in the years after implementation, and only 78% of the injured players wore a mouthguard during injury. An important outcome of the follow-up study was that the frequency and severity of orodental injury did not significantly differed between pre- and postimplementation of mandatory mouthguards, and prevalence of orodental injury remained high after implementation (15%). An explanation by the authors was that this follow-up study was performed too soon after implementation (16,17). With these results, it is unclear whether mandatory wearing of mouthguards is an effective strategy, and whether other countries worldwide should also implement mandatory wearing of mouthguards. It is therefore of great importance to evaluate the current situation at an international level to put the data into perspective.

The aim of this study was to determine the prevalence of orodental injury in Dutch and international field hockey, and to examine if there is a relationship between the number of orodental injuries and mouthguard usage.

## Material and Methods

This study has a cross-sectional study design by plotting a questionnaire in the Dutch field hockey competition and at the Master Hockey World Cup in Terrassa, Spain, to collect international data. A 19-item digital questionnaire was developed concerning orodental injury and mouthguard usage in field hockey. The questionnaire consisted of three consecutive parts: players characteristics; experience of orodental injuries; and mouthguard usage. If players had suffered from multiple injuries during their career, they were asked to provide details of the most recent injury. If players had not experienced orodental injury in hockey, they were automatically forwarded to the questions in section three relating to mouthguard usage. Inclusion criteria were: male and female hockey players > 14 years old. Goalkeepers, and those who were <14 years of age were excluded.

-Dutch data

In the period from the 1st of June to the 15th of August 2018, 55 Dutch hockey clubs were invited by email with the request to participate in the current study. Nineteen clubs agreed to participate and distributed the link to the digital questionnaire to their members.

In total, 1333 questionnaires were collected. Of these, 415 questionnaires were excluded; 45 questionnaires of goalkeepers, 230 questionnaires of players under the age of 14 years old, and another 140 questionnaires since only the part ‘players characteristics’ was completed. The remaining 918 questionnaires were analyzed. Twenty-five questionnaires had missing values only in the last part, about mouthguard usage, but were included because at least the first two parts of the questionnaire were completed and contained useful information for this study. Consequently, the number of respondents for different items of the questionnaire my vary.

-International data

International hockey players attending the Master World Cup in Terrassa, Spain, in the period from the 27th of July to the 5th of august 2018, were invited to fill in the questionnaire. All European countries were selectable in the questionnaire as well as a selection of non-European countries, who were officially participants of the tournament. Because of the low number of players per country, these were categorized into continents, and subdivided into European and non-European countries.

In total, 320 questionnaires were collected. Of these, 25 questionnaires were excluded: 9 questionnaires from goalkeepers, 3 from non-hockey players, and 13 questionnaires because only the section on ‘players characteristics’ was completed. The remaining 295 questionnaires were eligible for data analysis. However, some of the participants did not complete all the questions in the third part, but their data was included because of the relevance of the data they provided in the preceding questions. Consequently, the number for different statistical tests or Tables does vary.

-Ethical statement

The questionnaire was completely anonymous. Informed consent was automatically obtained when the participant began to complete the digital questionnaire. The present study did not include patients, participation did not bring along any risk, and participation was voluntarily. Ethical approval was not required, because the Dutch Medical Research on Humans Act (WMO) did not apply to this study. Nevertheless, the study was performed in accordance with the ethical principles as stated in the Declaration of Helsinki.

-Statistical analysis

Statistical analyses were performed using SPSS, version 25 (SPSS Corp. Released 2017. IBM SPSS Statistics for Windows, Version 25.0. Armonk, NY: IBM Corp). Standard descriptive statistics were used to describe the sample. We were using the using the Pearson Chi-Square test for comparison of categorical data. As significance level, alpha was set at 0.05.

## Results

-Dutch data

In total, 918 Dutch hockey players completed the questionnaire. Demographic and professional characteristics of the respondents are presented in Table 1. From all participants, 187 players (20%) suffered from orodental injury at least once in their career. There was no significant difference in prevalence of orodental injury between men and women (*p*=0.11). Players who suffered from orodental injury last season are represented as the incidence of orodental injury, which is 2,8% from the total number of players. Orodental injuries occurred in 105 players (56%) during a match and in 105 players (56%) was caused by a hockey ball. In 113 players (60%), the injury involved teeth. One-hundred-and-nineteen players (64%) had to withdraw from the training/match after injury, and 143 players (79%) requested treatment.

**Table 1:**
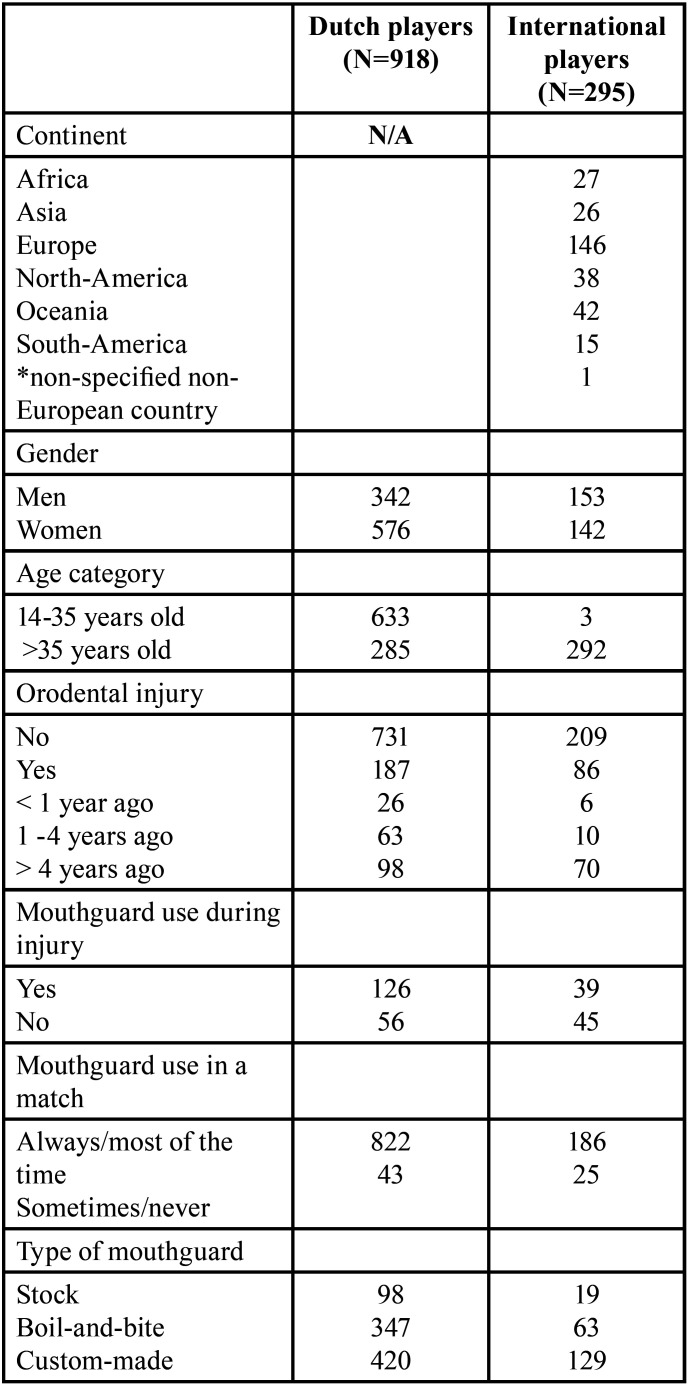
Characteristics of included players in the Dutch and international questionnaire.

**Figure 1:**
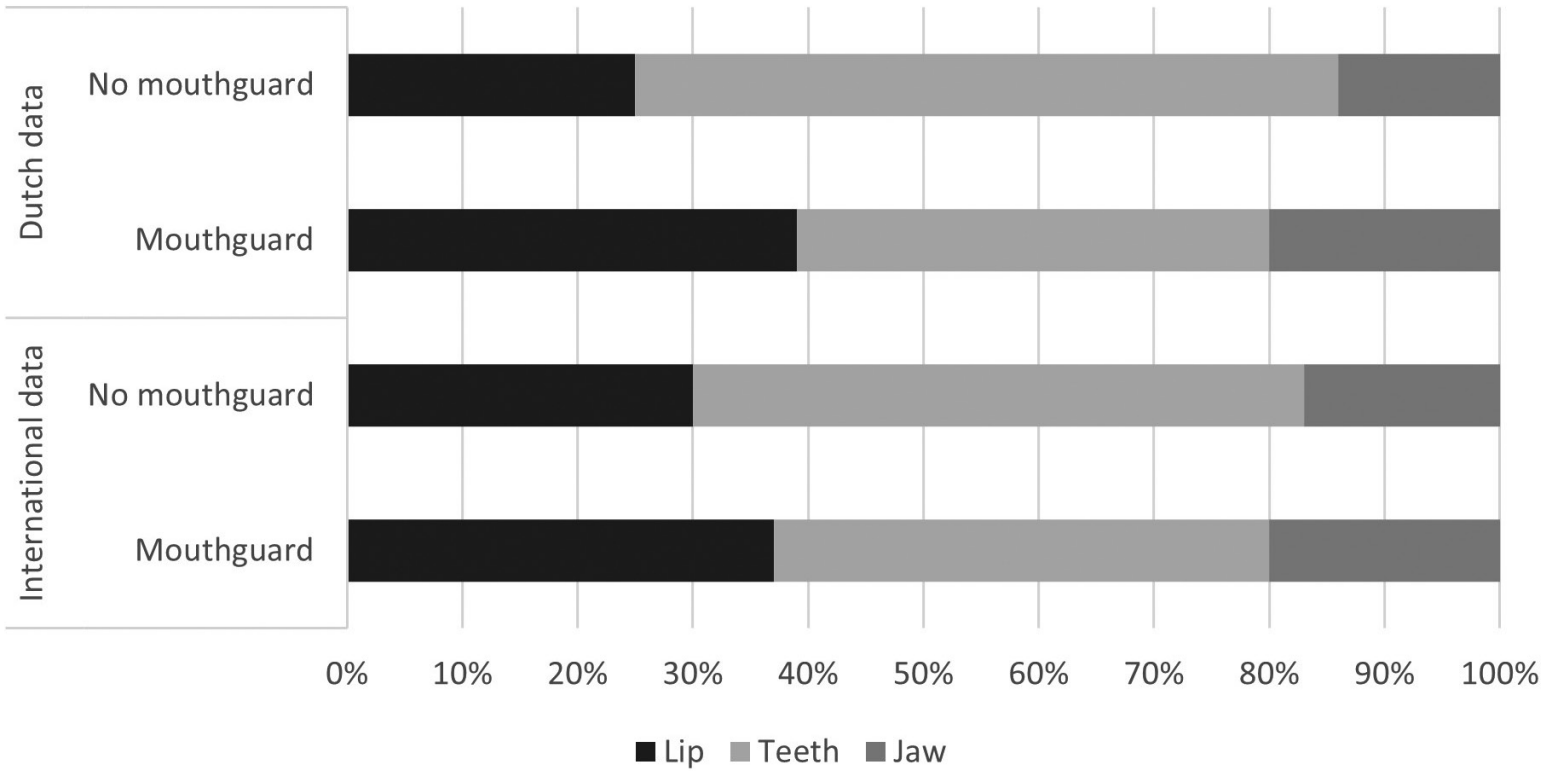
Percentage per injured facial site in relation to mouthguard use during injury for Dutch and international players.

**Figure 2:**
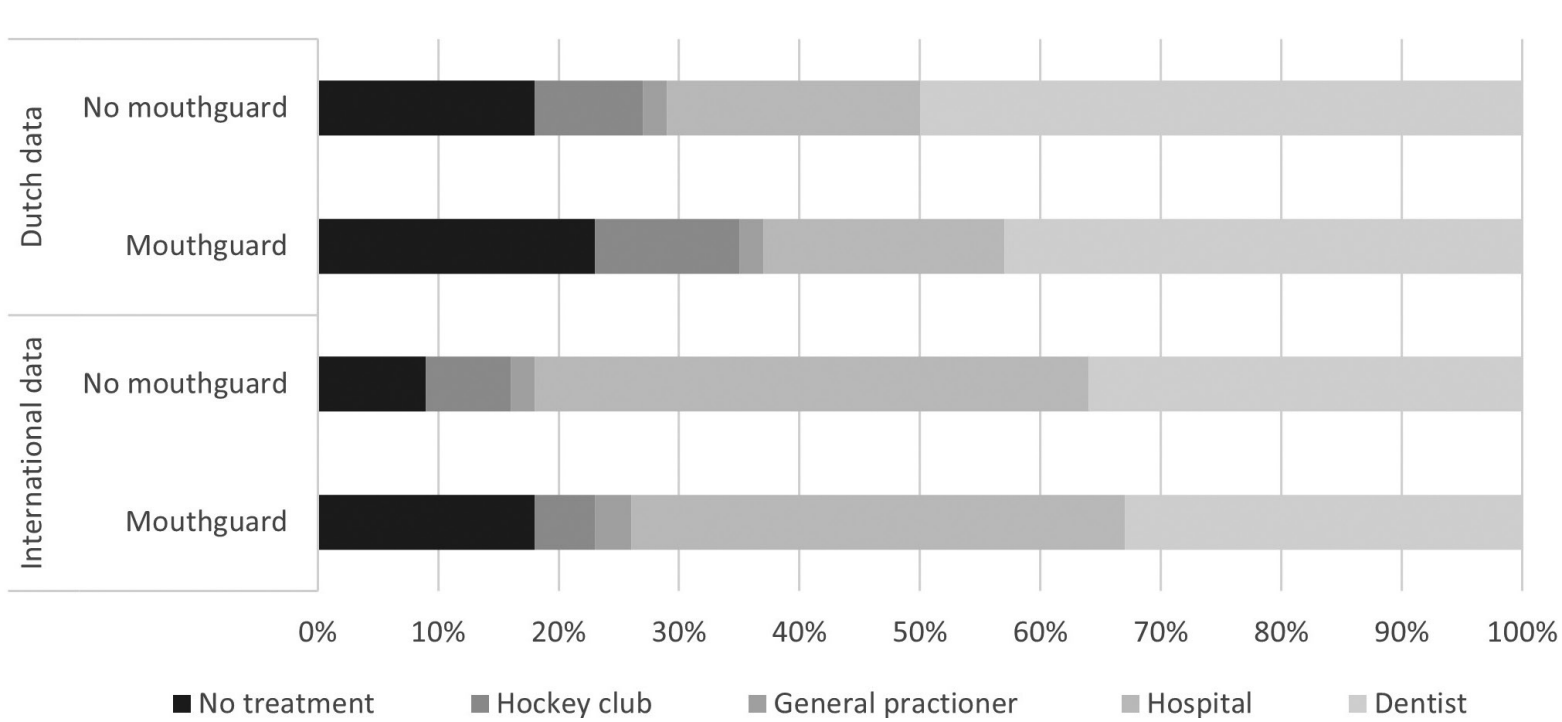
Percentage per type of requested treatment in relation to mouthguard use after injury for Dutch and international players.

Of all players, 757 players (96%) were in the possession of a mouthguard. Eight-hundred-twenty-two players (95%) were frequently (always and most of the time) using their mouthguard during matches. Of all injured players, 126 players (69%) wore a mouthguard during the event. Figure 1 shows the injured facial site in relation to whether the player wore a mouthguard or not during this injury. Players with mouthguard suffered significantly more often from lip and teeth injuries compared to players without mouthguard (lip injury *p*=0.01, teeth injury *p*= 0.01). There was no significant difference between wearing a mouthguard or not and whether players requested treatment as a result of an injury (*p*=0.43) (Fig. 2).

Custom-made type mouthguards were worn by 420 players (49%). For all types of mouthguards, the highest number of complaints (33%) related to the negative effect of mouthguard usage on communication on the hockey field. Players using a custom-made mouthguard had significantly fewer complaints than those using stock mouthguards and boil-and-bite mouthguards (*p*<0.01). Six-hundred-forty-eight (75%) players believe that their currently used mouthguard could be improved with regard to comfort and protection. From the players with this opinion, 385 players (44%) wear a custom-made mouthguard, 279 players (43%) a boil-and-bite mouthguard and 84 players (13%) a stock mouthguard.

Seven-hundred-seventy-seven players (90%) felt safer wearing a mouthguard.

-International data

In total, 295 international field hockey players completed the questionnaire. Demographic and professional characteristics of the respondents are presented in Table 1. We collected data of players from 21 different countries: 146 questionnaires were collected from European countries, 26 from Asian countries, 27 from African countries, 42 from Oceanian countries, 38 from North-American countries, 15 from South-American countries, and 1 questionnaire from a person who did not specify his/hers non-European country.

In total, 86 international players (29%) reported that they had suffered from orodental injury at least once in their career. There was no significant difference in prevalence of orodental injury between men and women (*p*=0.36). The prevalence of injury in European players was 28%, and in non-European players 30%. There was no significant difference between European and non-European players (*p*=0.66). Of all the players who sustained an injury, in 6 players (7%) this injury occurred last season. Orodental injuries occurred in 60 players (71%) during matches, and in 47 players (56%) was caused by a hockey ball. In 54 players (63%), injury was to teeth. Thirty-eight players (45%) had to withdraw from the activity after injury and 73 players (87%) requested treatment.

Of all players, 174 players (69%) were in the possession of a mouthguard. Hundred-eighty-six players (79%) were using their mouthguard frequently (always and most of the time) during matches. There was no significant difference in the comparison of mouthguard usage during matches between European (92%) and non-European (85%) players (*p*=0.16). However, mouthguard usage was significantly higher among European payers than by players from Asia (*p*<0.01) and Africa (*p*<0.01).

Of all injured players, 39 players (46%) wore a mouthguard during the event. Figure 1 shows the injured facial site in relation to whether the player wore a mouthguard or not during this injury. There were no significant differences between wearing a mouthguard or not and the location of facial injury (lip injury *p*=0.30, teeth injury *p*= 0.34). There was no significant difference between wearing a mouthguard or not and whether players requested treatment as a result of an injury (*p*=0.22) (Fig. 2).

Custom-made type mouthguards were worn by 129 players (61%). Players using a custom-made mouthguard had significantly fewer criticisms in general than those using stock mouthguards (*p*=0.03) or boil-and-bite mouthguards (*p*<0.01). There was no significant difference in the level of criticism relating to stock and boil-and-bite mouthguards (*p*=0.41). The highest number of complaints (34%) related to the negative effect of mouthguard usage on the communication on the hockey field. Hundred-forty-six players (69%) believed that their currently used mouthguards could be improved in terms of comfort and protection. From the players with this opinion, 80 players (55%) wear a custom-made mouthguard, 53 players (36%) a boil-and-bite mouthguard and 13 players (9%) a stock mouthguard.

Two-hundred-four players (97%) players felt safer wearing a mouthguard.

## Discussion

In this study, the prevalence of orodental injury in the Dutch and international field hockey competition was determined, together with the relation between the number of orodental injuries and mouthguards usage.

The results of this study showed that the prevalence of orodental injury among hockey players is high: 20% in Dutch field hockey, and 29% in international field hockey. This prevalence is more or less in accordance to previously performed studies. For example, Hendrick *et al*. examined the prevalence of orodental injury among 110 female elite field hockey players in England; 19% of these players suffered from orodental injury at least once in their career (18). Zamora-Olave *et al*. found an even higher prevalence of orodental injury in field hockey of 50%, which was examined in 325 players of two hockey clubs in Spain. According to the author, the high prevalence of orodental injury could partly be explained because TMD was taken into account, unlike other studies (19). Tinoco *et al*. examined dental injury among 135 young elite field hockey players at the pre-Olympic competition, and found a prevalence of 27%. In this study, the most frequent type of injury was lip laceration (56%), followed by dental injury (31%). The authors also reported that more than half of the players did not wear a mouthguard, as an explanation for these high numbers of injury (20). However, our results showed that despite wearing a mouthguard these two types of orodental injury, lip laceration and dental injury, remain high. Wearing a mouthguard also did not affected requested medical treatment.

All the studies above found high prevalence of orodental injury in combination with a high usage of mouthguards, which is in consistent with the results of our study. The combination of these results could imply that the current mouthguards are not sufficiently effective in protecting players from orodental injuries. A previous study by the current authors tested dental models with applied mouthguards on maximum impact heights and speeds. The results of that study also showed that mouthguards (all types) may not provide the right level of protection against the high forces encountered during hockey to prevent teeth injury (21).

Sarao *et al*. showed that the compliance of wearing a mouthguards depends on the level of comfort, and communication on the field (22). The majority of the players in the present study reported to wear a custom-made mouthguard, which was significantly less associated with criticism compared to the other types of mouthguards. However, there is still some difficulty with communication on the hockey field while wearing a mouthguard. This explains why 69-75% of the respondents supports the development of a better, more comforTable mouthguard in the future.

The strength of the present study is that there are no studies that have examined the prevalence of orodental injury and mouthguard usage in such a large and diverse sample of hockey players. Also at this moment, mouthguards are only recommended by the Fédération Internationale de Hockey (FIH) (23), and to our knowledge the Netherlands is the only country where mouthguards are mandatory. The results of our study, conducted independently from any regulatory board or government, may contribute to further evidence-based decisions with regards to whether mouthguards in hockey should be mandatory or not.

One of the limitations in this study was the relatively low number of participants in the international questionnaire, compared to the Dutch questionnaire, which resulted in the decision to analyze the international responses at a continent level instead of country level to enable more reliable comparisons. Furthermore, in both questionnaires, the severity of the injuries is derived from the answer whether the player requested treatment for the sustained injury. However, it is possible that some players did not go to the most relevant health professional for treatment. The players in this study may have attended a hospital more frequently, creating an overestimation with regard to the severity of the injury. The actual severity of any injury can only be determined when a clinical diagnosis is made by a health professional.

## Conclusions

The prevalence of orodental injury in field hockey remains high, despite frequent mouthguard usage. Players who sustained injury whilst wearing a mouthguard did not requested less treatment compared to those not wearing a mouthguard. The effectiveness of the currently used field hockey mouthguards should be further examined in order to reduce orodental injury in this sport.
